# Supplementary material for: Memory T cells targeting oncogenic mutations detected in peripheral blood of epithelial cancer patients
Source: Nat Commun. 2019 Jan 25;10:449. doi: 10.1038/s41467-019-08304-z (PMC6347629; doi:10.1038/s41467-019-08304-z)
Supplement: Supplementary file 1 — Description of Additional Supplementary Files [file 41467_2019_8304_MOESM1_ESM.docx]

**Supplementary data**

**Title:** Supplementary data 1.
**Description:** Patient 4217: Somatic mutations identified by whole-exome sequencing.

**Title:** Supplementary data 2.
**Description:** Patient 4213: Somatic mutations identified by whole-exome sequencing

**Title:** Supplementary data 3.
 **Description:** Patient 4148: Somatic mutations identified by whole-exome sequencing

**Title:** Supplementary data 4.
**Description:** Patient 4171: Somatic mutations identified by whole-exome sequencing

**Title:** Supplementary data 5.

**Description:** Patient 4238: Somatic mutations identified by whole-exome sequencing

**Title:** Supplementary data 6.
**Description:** List of primers used in this study
