# Supplementary material for: Memory T cells targeting oncogenic mutations detected in peripheral blood of epithelial cancer patients
Source: Nat Commun. 2019 Jan 25;10:449. doi: 10.1038/s41467-019-08304-z (PMC6347629; doi:10.1038/s41467-019-08304-z)
Supplement: Supplementary file 2 — Supplementary Information [file 41467_2019_8304_MOESM2_ESM.pdf]

Supplementary Materials for

Memory T cells targeting oncogenic mutations detected in peripheral blood of epithelial cancer patients

Authors:
Gal Cafri,[1#] Rami Yossef,[1#] Anna Pasetto,[1] Drew C. Deniger,[1] Yong-Chen Lu,[1] Maria Parkhurst,[1] Jared J. Gartner,[1] Li Jia,[1] Satyajit Ray,[1] Lien T. Ngo,[1] Mohammad Jafferji,[1] Abraham Sachs,[1] Todd Prickett,[1] Paul F. Robbins [1] and Steven A. Rosenberg [1]*

Affiliations:

[1]Surgery Branch, National Cancer Institute, National Institutes of Health, Bethesda, MD 20892, USA

[#] **These authors contributed equally to this work**

* **Corresponding to:** Steven A. Rosenberg, M.D., Ph.D. **-** sar@nih.gov

This file includes:

Supplementary Figures 1 to 9

Supplementary Tables 1 to 7

Supplementary notes 1-2

Supplementary figures

Supplementary Figure 1

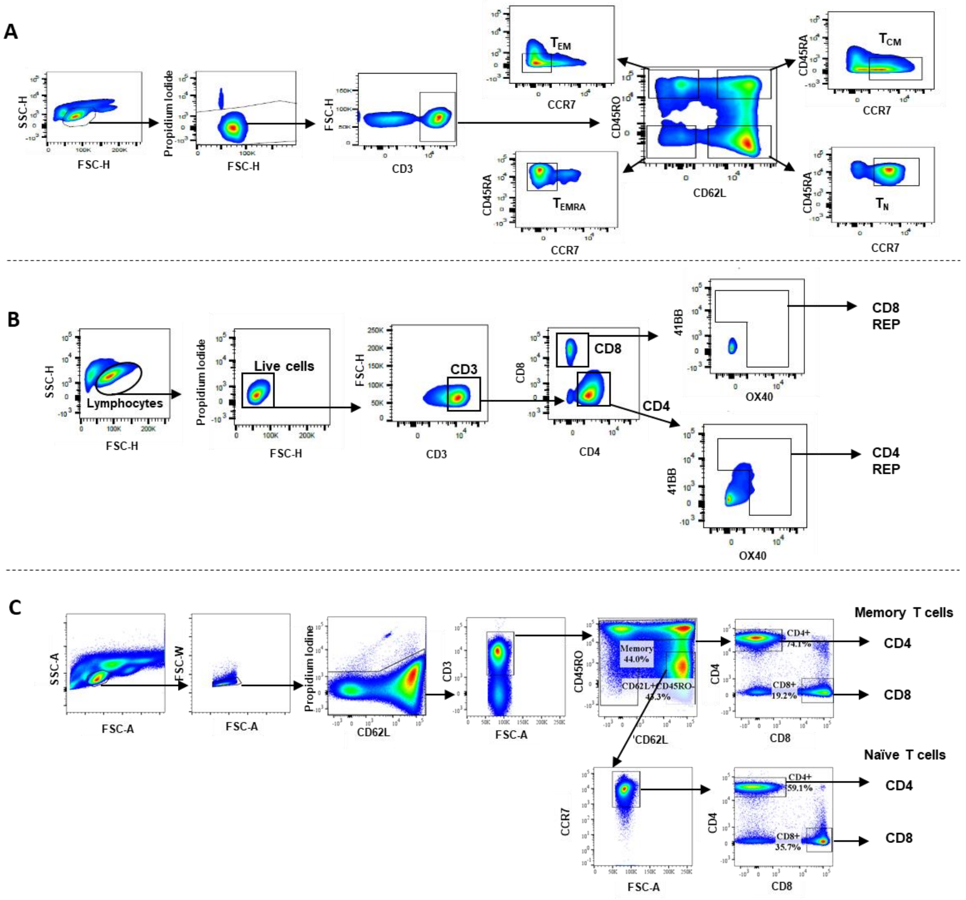

Supplementary Figure 1. Gating strategies used for cell sorting (A) For data presented in Fig.1C/D, Fig. 3 and supplementary figure 3. Frozen PBMC were thawed and rested overnight in complete T cell medium without cytokines. Cells were collected and stained using antibodies against CD3, CD8, CD62L, CD45RO, CCR7 and CD45RA. For sorting cells were gated based on CD45RO and CD45RA following by second gate based on CD45RA and CCR7. Cells were than sorted according to the gate set on Fig.A from the CD45RA/CCR7 gate. (**B)** Gating strategy for day 10 re-stimulation and enrichment for 41BB and OX40 positive cells. **(C)** For data presented in Fig. 3, 4 and supplementary figure 8. Frozen PBMC were thawed and rested overnight in complete T cell medium without cytokines. Cells were collected and stained using antibodies against CD3, CD8, CD62L, CD45RO, and CCR7. For sorting cells were gated based on CD45RO and CD45RA following by second gate based on CCR7 to sort Naïve cells.

Supplementary Figure 2

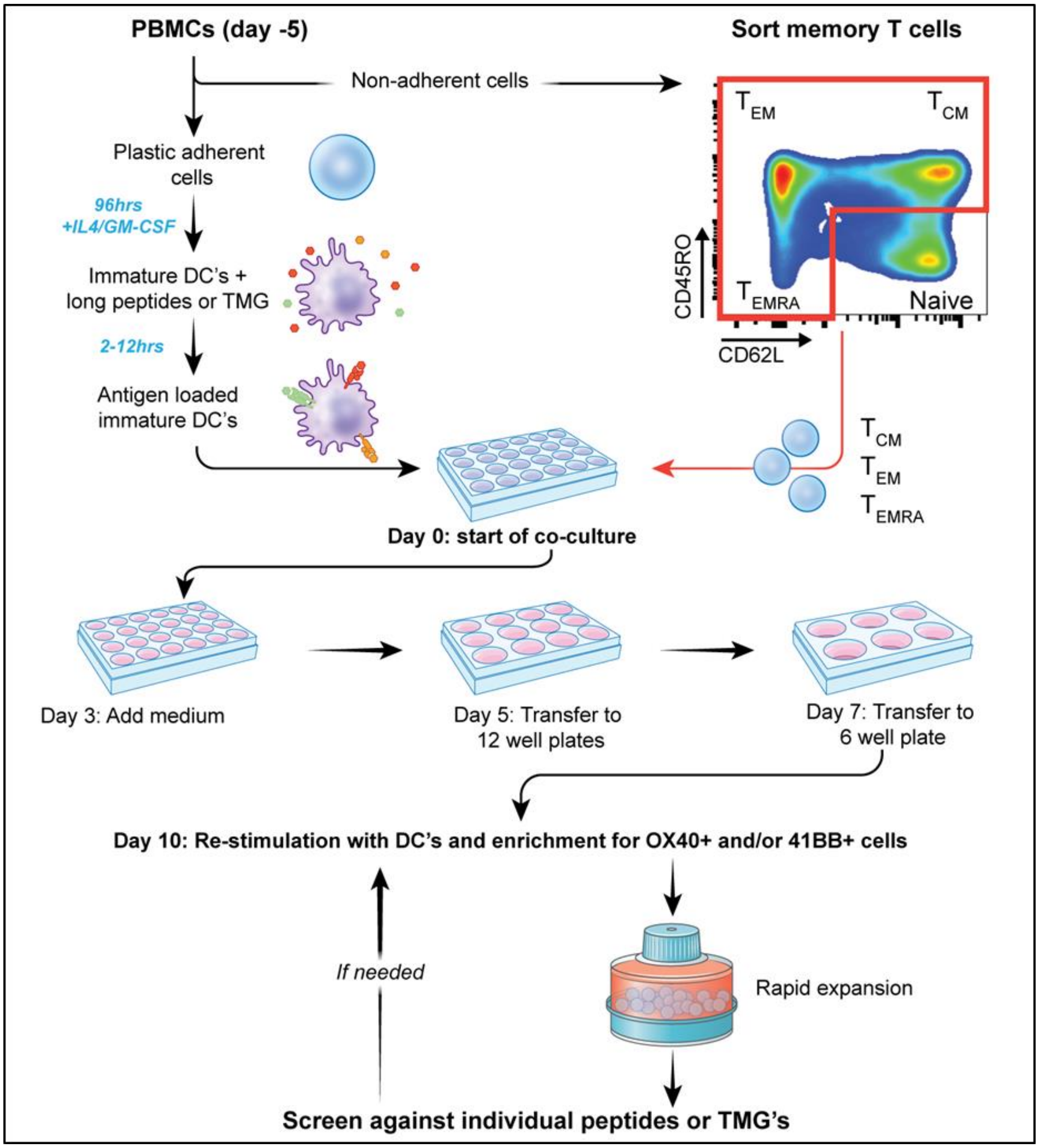

Supplementary Figure 2. Overview of the IVS method used for the isolation of neoantigen specific T cells from peripheral blood

Supplementary Figure 3

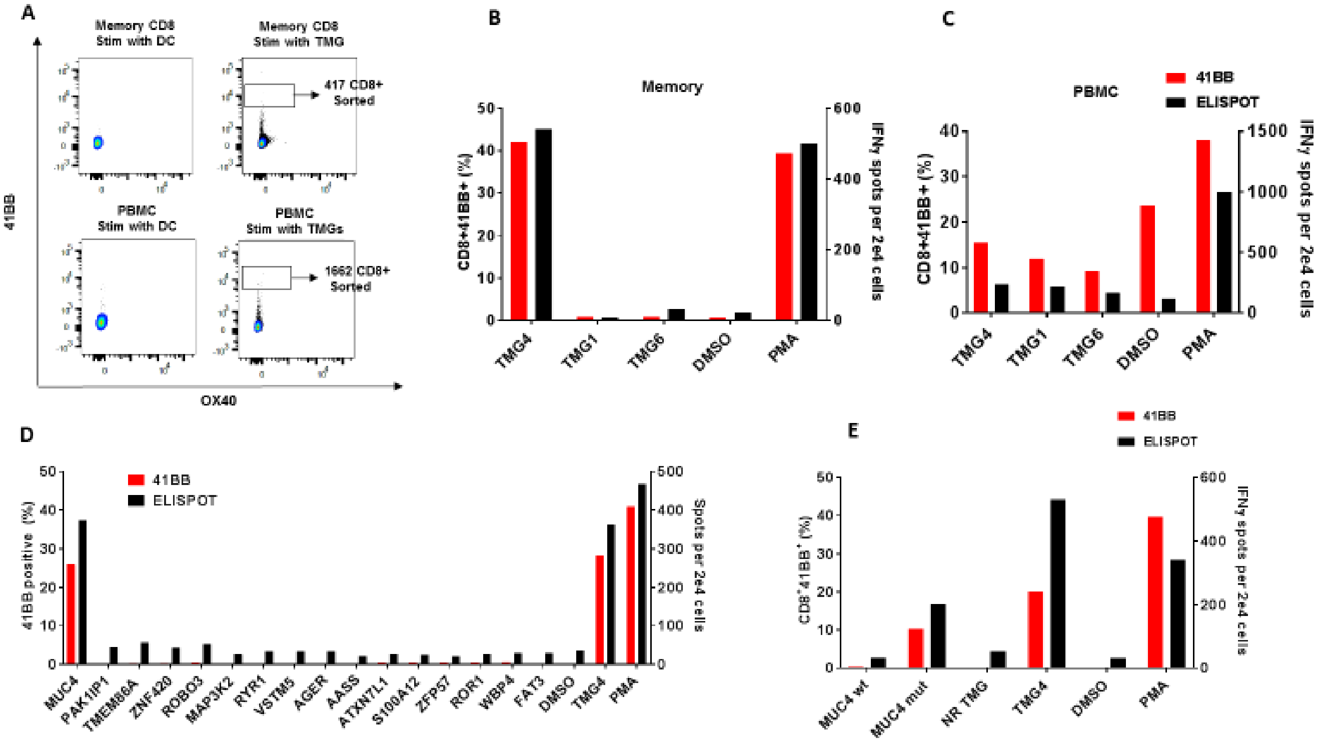

Supplementary Figure 3. Identification of T cells specific for mut MUC4 in the PBMC of a metastatic colon cancer patient. (A) Memory and Bulk CD8 cells were co-cultured with autologous DCs transfected with 3 TMGs for 18 hours. Activated T Cells were stained for CD3, CD8, 4-1BB, and OX40 and sorted based on 4-1BB and OX40 expression to enrich for neoantigen reactive cells. **(B, C)** Memory and bulk PBLs T cells isolated in **A** were co-cultured with autologous DCs transfected with TMGs 1, 4 and 6 for 18 hours, stained with CD3, CD8, and 4-1BB and analyzed for surface expression of 4-1BB as a marker for T cell activation. **(D)** Memory CD8 cells isolated in **A** were co-cultured for 18 hours with autologous DCs that were individually pulsed with the mutated peptides encoded by TMG4 and tested either by flow cytometry for 4-1BB expression or IFNγ-secretion using ELISPOT assay. **(E)** Memory CD8 cells isolated in **A** were co-cultured for 18 hours with autologous DCs that were loaded with WT or Mut MUC4 LP and transfected with TMG4. Cells were tested for antigen recognition by flow cytometry for 4-1BB expression or IFNγ-secretion using ELISPOT assay.

Supplementary Figure 4

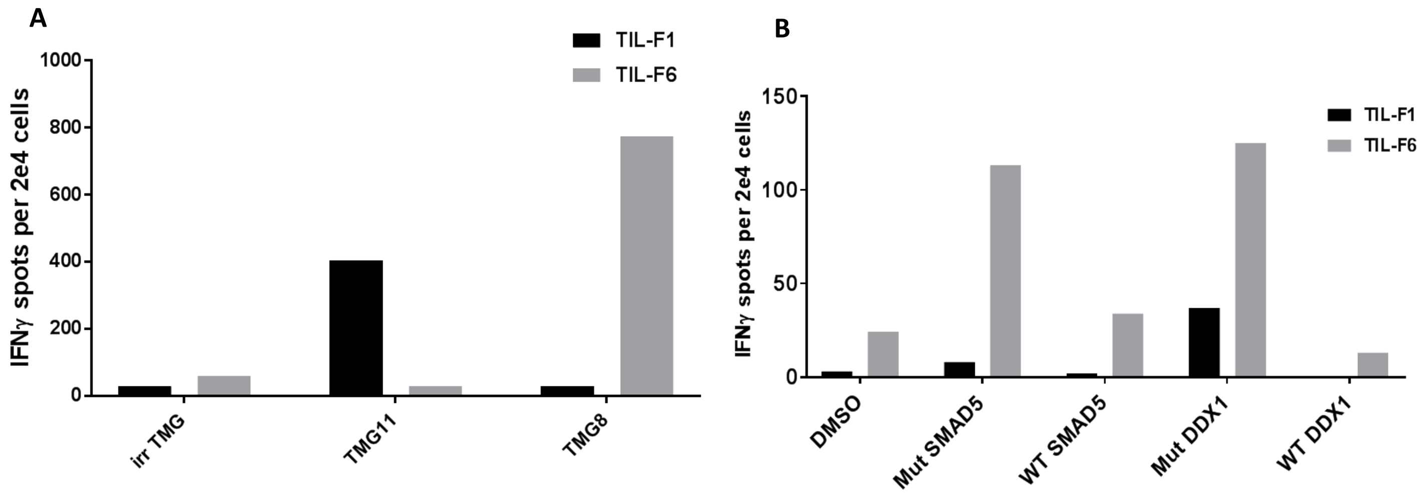

Supplementary Figure 4. Identification of mutation-specific T cells in a patient with metastatic colonl cancer (4213). (A) Two different TIL cultures were co-cultured with autologous DCs transfected with the indicated TMG construct encoding the various putative mutations identified by whole-exomic sequencing. T-cell responses were measured the next day by IFN-γ ELISPOT assay **(A)**. To exclude WT recognition, TIL cultures were co-cultured with autologous DCs loaded with the indicated peptides, T-cell responses were measured the next day by IFN-γ ELISPOT assay **(B)**

Supplementary Figure 5

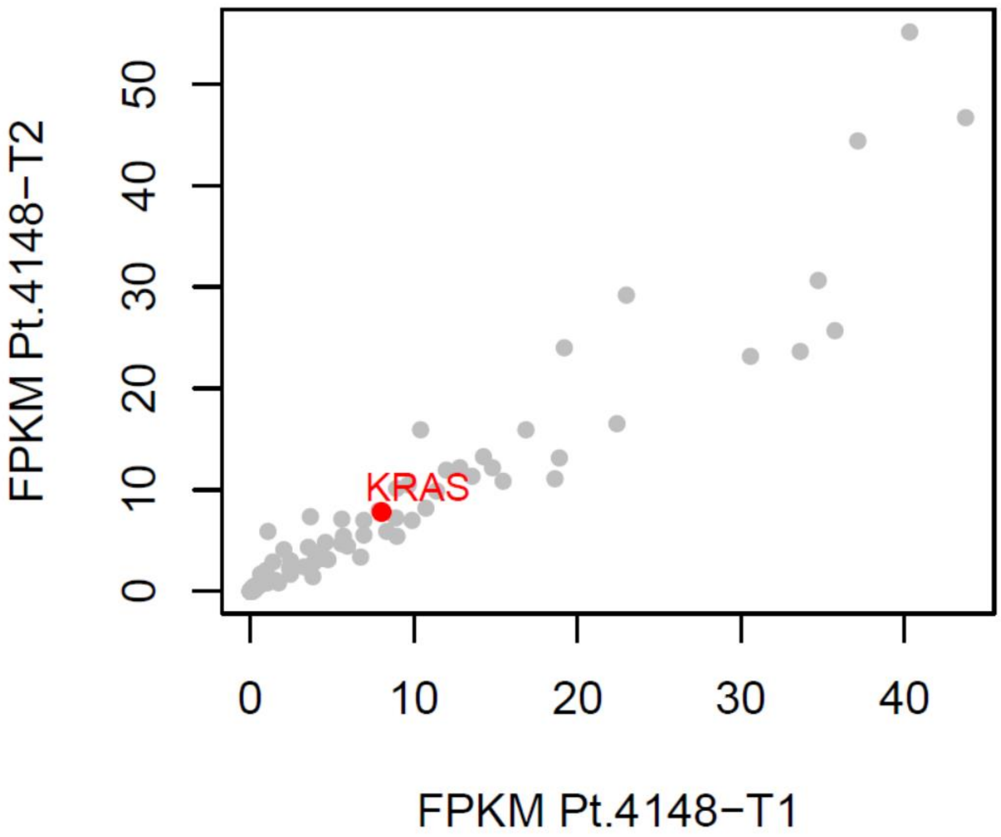

Supplementary Figure 5. Shared $KRAS^{G12V}$ expression in two excised lesions from Pt.4148. RNAseq showing shared mutations between two excised tumors, *KRAS* mutated transcript is presence in both excised lesions at the same levels, FPKM=~8.5.

Supplementary Figure 6

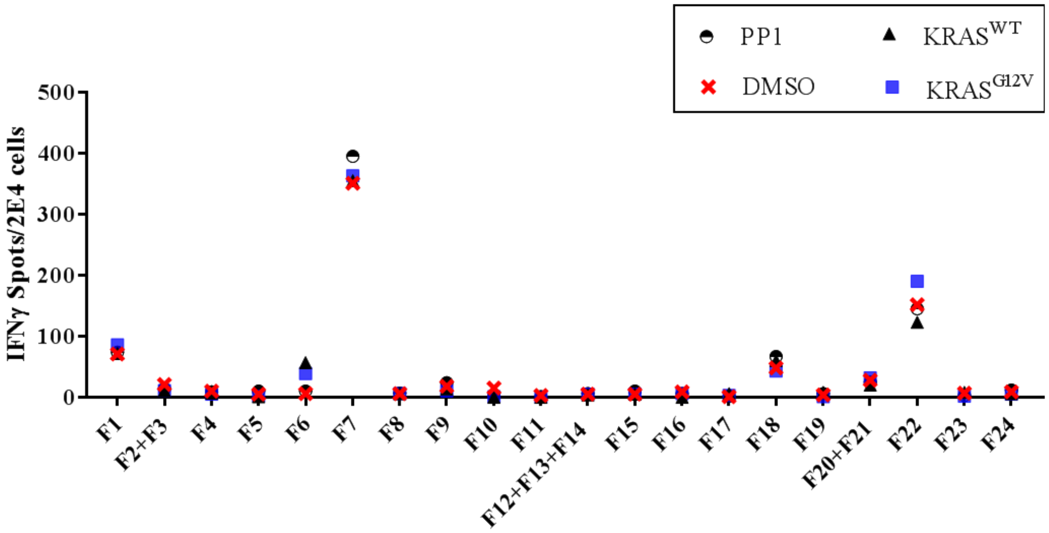

Supplementary Figure 6. TIL fragments from Pt.4148 did not show reactivity against $KRAS^{G12V}$. TIL fragments from Pt. 4148 were grown ex vivo following tumor excision and co-cultured with autologous DCs pulsed with $KRAS^{G12V}$ and $KRAS^{WT}$ and IFNγ secretion was assessed by ELISPOT.

Supplementary Figure 7

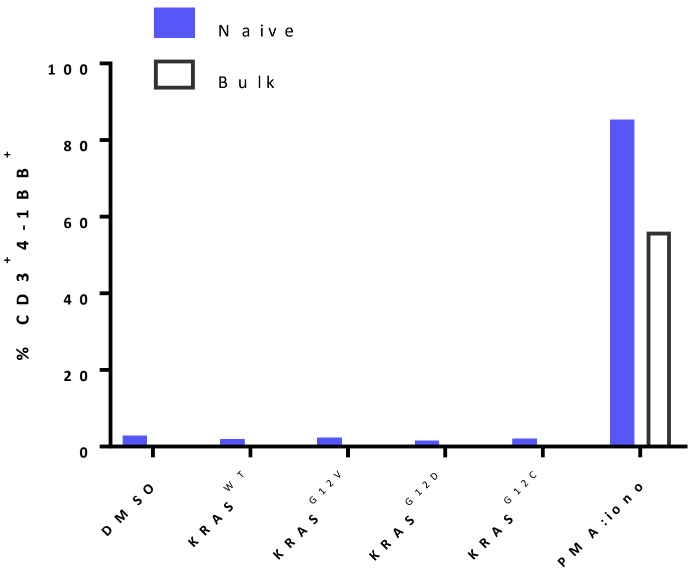

Supplementary Figure 7. Pt.4148 bulk and naive CD8 subsets did not show reactivity against mutated KRAS. 4-1BB-enriched CD8 bulk and naive T cells were expanded, and their reactivity was tested against autologous DCs pulsed with the indicated peptides.

Supplementary Figure 8

A

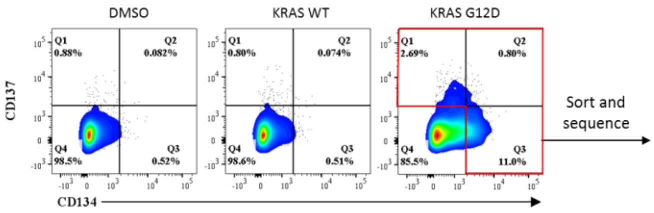

B

TCR	CDR3β		CDR3α
TCR1	CASRGTEGTEAFF	TRBV9-1	TRAV8-1
TCR2	CASSEALSGGAFGGELFF	TRBV6-1	TRAV41-1
TCR3	CASSENLAGAANTGELFF	TRBV6-1	TRAV41-1
TCR4	CASSLQGAMNTEAFF	TRBV5-4	TRAV38-2
TCR5	CASSPRTGGTTIGEQFF	TRBV19	TRAV3-1
TCR6α1<br>TCR6α2	CASSVSLTQYGYTF	TRBV9-1	TRAV12-2
TCR7	CAWSRGGNQPQHF	TRBV30-1	TRAV23
TCR8	CASSNSGAAVDTDTQYF	TRBV5-1	TRAV8-3
TCR9	CSVDERGGTHGYTF	TRBV29-1	TRAV12-2
TCR10	CASSRGLATTDTQYF	TRBV7-7	TRAV4
TCR11α1<br>TCR11α2	CSALGGGNTGELFF	TRBV20-1	TRAV1-1

C

D

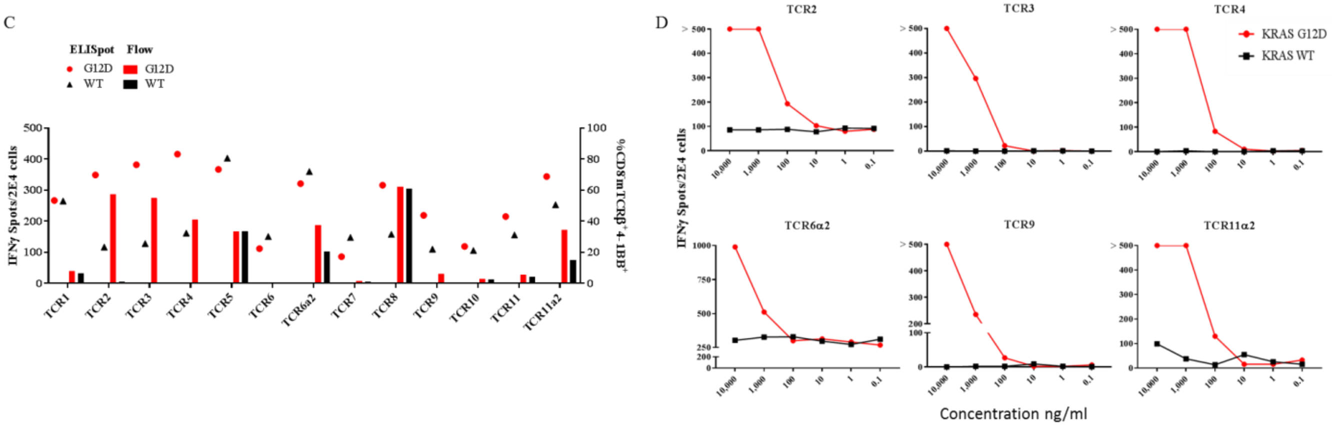

Supplementary Figure 8: Isolation of T-cell receptors from PBMC targeting KRAS^{G12D}. (A) Upregulation of T-cell activation markers of 4-1BB^{+} and/or OX40^{+}-enriched CD4 memory T cells following co-incubation with autologous DCs pulsed with mutated and WT 24mer peptides. **(B)** Following co-culture with DCs pulsed with mutated peptides, memory T cells recognizing the mutated peptides were FACS-sorted into 96-well PCR plates for scPCR based on T-cell activation markers. 11 unique TRBV and 13 TRAV were obtained. **(C)** IFNγ-secretion ELISPOT assay of retrovirally-transduced allogeneic PBMCs following co-incubation with DCs pulsed with mutated and WT peptides. **(D)** IFNγ secretion of TCR-transduced cells with the reactive TCRs co-incubated for 18hrs with autologous DCs pulsed with the indicated concentrations of mutated and WT. Representative results of at least 3 experiments.

Supplementary Figure 9

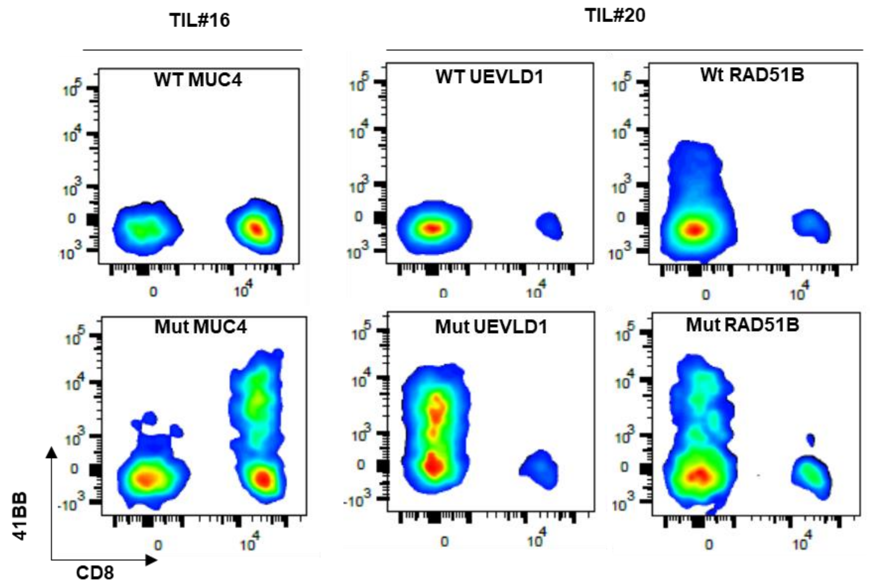

Supplementary Figure 9. Identification of mutation-specific T cells in a patient with metastatic colon cancer (4217). Two different TIL cultures were co-cultured with autologous DCs loaded with the indicated peptides. T-cell responses were measured the next day by 41BB upregulation.

Supplementary tables

Supplementary Table 1. Patient 4213 TMG6 and TMG8 antigens list

TMG	Mutated gene	Sequence
**TMG-8**	NEDD4	SNVVQMKLTPRQSALAPLIKENVQS
	SPTBN2	MDAQERPRDVSSVDLVIKNQQGIKA
	SEC22C	GSAEGCDFSIQLVDILKEAEERQSQ
	BAI3	KGRKGMVDWARNPEDRVVIPKSIFT
	PTCH2	SLPCARWNLAHFTRYQFAPLLLQSH
	EYS	CPKGSSSQNGETGVSEFSLVPCQNG
	SMAD5	SSRDVQPVAYEEPKHKHWCSIVYYE
	MUC4	ASSVSTGHATPLHVTDASSVSTGHA
	PCDHGA12	TVAVADSIPQVLVDLGSLESPANSE
	SIL1	DINTNTYTSQDLRSALAKFKEGAEM
	IRX1	NYSAFLPYAADLRLFSQMGSQYELK
	ZNF775	PECGRRFSQKPNVTRHLRNHTGERP
	SVEP1	GGHIQYQCFPGYTLHGNSSRRCLSN
	ASH1L	SDLEMEPDKKITMRNNGQLMKTIIR
	HTR1B	KLHTPANYLIASPAVTDLLVSILVM
**TMG-6**	TYROBP-2	QHPSPPAATRKQCITETESPYQELQ
	C19orf52	AHLRVVGPQQLHYETNERLFDEKYK
	SMNDC1	WQQFNNRAYSKNRKGQVKRSIFASP
	GABRP	FLHEVTVGNRLILLFSNGTVLYALR
	HEBP2	LASILREDGKVFGEKVYYTAGYNSP
	TDRP,ERICH1	EHCTMPPGVLSRRKAKKIRRGKREG
	DNAH9	VCYFMERLMARQWPVMLVGTAGTGK
	WDR24	NLLATAATNGVVATWNLGRPSRNKQ
	ZNF559	PFTESSYLTQHLGTHSRVLPIEHKK
	SPATA5	GPQSDSDTDAQRVAFEQSSMETSSL
	PCLO	SCTAQQPATTLPDDRFGYRDDHYQY
	TYROBP-3	RGRGAAEATRKQCITETESPYQELQ
	MIB2-1	LDLAAEGRVLKALRAAPSASECARRMKKCIRCQVVVSKKLRPDG S
	PPP2R3A	CTGTCHTFTHGIHCIVVHHSVCADL
	CSGALNACT1	NFTFIQLNGEFSQGKGLDVGARFWK

Supplementary Table 2. TCR-VB sequences of neoantigen reactive TIL

Histology	Patient	Target Neoantigen	TIL phenotype	TCR-VB CDR3 sequence
**Colon**	4213	DDX1	CD8	CASGVAESSYEQYF
		SMAD5	CD8	CASGLVSGQGAGVTEAFF
**Ovarian**	4046	USPX	CD4	CASSSGTSADTQYF
**NSCLC**	4134	GRB7	CD8	CASSQGSYEQYF
**Endometrial**	4148	$KRAS^{G12V}$	CD4	CSAREGAGGMGTQYF

Supplementary Table 3. PBL derived TCR-VB sequences of cloned TCRs

Histology	Patient	Target Neoantigen	CD4/CD8	CDR3β sequence
**Colon**	4213	SMAD5	CD8	CVSGLVSGQGAGVTEAFF
**Endometrial**	4148	$KRAS^{G12V}$	CD8	CASSLTSGGFDEQFF
**Rectal cancer**	4171	$KRAS^{G12D}$	CD4	CASSVTGGSYPNTEAFF
**Sigmoid colon adenocarcinoma**	4238	$KRAS^{G12D}$	CD4	CASSEALSGGAFGGELFF
				CASSENLAGAANTGELFF
				CASSLQGAMNTEAFF
				CASSVSLTQYGYTF
				CSVDERGGTHGYTF
				CSALGGGNTGELFF

Supplementary Table 4. Mutated KRAS exome and RNA sequencing across 21 cancer patients

Patient	Number of metastases tested for KRAs mutation	Number of metastases with DNA mutation	Number of metastases with RNA mutation	Gene annotation	Clonality
**1**	2	2	2	NM_033360:exon2:c.G35A:p.G12D	Clonal in all exome samples
**2**	2	2	2	NM_033360:exon2:c.G34C:p.G12R	Clonal in -2 subclonal in -1
**3**	3	3	3	NM_033360:exon2:c.G35T:p.G12V	Clonal in all exome samples
**4**	2	2	2	NM_033360:exon2:c.G34T:p.G12C	Clonal in all exome samples
**5**	3	3	3	NM_033360:exon2:c.G35C:p.G12A	Clonal in all exome samples
**6**	3	3	2*	NM_033360:exon2:c.G34T:p.G12C	Clonal in 1 subclonal in 2
**7**	2	2	2	NM_033360:exon2:c.G35A:p.G12D	Clonal in all exome samples
**8**	2	2	2	NM_033360:exon2:c.G34T:p.G12C	Clonal in all exome samples
**9**	2	2	2	NM_033360:exon2:c.G35A:p.G12D	Clonal in all exome samples
**10**	3	3	3	NM_033360:exon4:c.G436A:p.A146T	Clonal in all exome samples
**11**	2	2	2	NM_033360:exon2:c.G34C:p.G12R	Clonal in all exome samples
**12**	3	3	3	NM_033360:exon2:c.G34C:p.G12R	Clonal in all exome samples
**13**	2	2	2	NM_033360:exon2:c.G35T:p.G12V	Clonal in all exome samples
**14**	3	3	3	NM_033360:exon2:c.G34T:p.G12C	Clonal in all exome samples
**15**	2	2	2	NM_033360:exon2:c.G35T:p.G12V	Clonal in all exome samples
**16**	2	2	2	NM_033360:exon2:c.G38A:p.G13D	Clonal in all exome samples
**17**	3	3	3	NM_033360:exon2:c.G35A:p.G12D	Clonal in all exome samples
**18**	2	2	2	NM_033360:exon2:c.G35T:p.G12V	Clonal in all exome samples
**19**	2	2	1**	NM_033360:exon2:c.G35T:p.G12V	Clonal in all exome samples
**20**	2	2	2	NM_033360:exon2:c.G35A:p.G12D	Clonal in all exome samples
**21**	3	3	3	NM_033360:exon2:c.G38A:p.G13D	Clonal in all exome samples

*Strong 3' bias in the RNA sequencing for 2 met leading to lower coverage of the 5' end

**Very low RNA seq coverage in 1 met that probably lead to lack of detection

Supplementary Table 5. HLA binding predictions for pt. 4148

Peptide	%Rank	Binding level	Length
MTEYKLVVVG	48.25		**10mer**
TEYKLVVVGA	42.5714		
EYKLVVVGAD	86.6667		
YKLVVVGADG	95		
KLVVVGADGV	33.4		
LVVVGADGVG	83.3333		
VVVGADGVGK	0.3308	Strong binder	
VVGADGVGKS	6.9675		
VGADGVGKSA	40.25		
GADGVGKSAL	33.6667		
ADGVGKSALT	65		
DGVGKSALTI	51.1765		
GVGKSALTIQ	10.8737		
VGKSALTIQL	43.8333		
GKSALTIQLI	37.7273		
MTEYKLVVV	20.5647		**9mer**
TEYKLVVVG	37.6364		
EYKLVVVGA	31.8947		
YKLVVVGAD	96.6667		
KLVVVGADG	48.75		
LVVVGADGV	25.3333		
VVVGADGVG	52.6471		
VVGADGVGK	0.2142	Strong binder	
VGADGVGKS	27.5		
GADGVGKSA	23.7963		
ADGVGKSAL	43		
DGVGKSALT	61.6667		
GVGKSALTI	10.6188		
VGKSALTIQ	14.9626		
GKSALTIQL	27.9333		
KSALTIQLI	11.3813		

Supplementary Table 6. **Summary of neoantigen isolation from different T cell populations used for IVS**

		Detected in		
Patient	Neoantigen	Memory	Naïve	Bulk PBMC
4213	$SMAD5^{P268inPKH}$	✓	✕	✕
4217	$MUC4^{R4435S}$	✓	✕	✕
4171	$KRAS^{G12D}$	✓	✕	✓ *
4148	$KRAS^{G12V}$	✓	✕	✕
4238	$KRAS^{G12D}$	✓	✕	✕

* $KRAS^{G12D}$–reactive TCR detected in sorted bulk $CD4^+$. Identical to TCR isolated from the memory $CD4^+$ subset.

Supplementary Table 7.

The therapeutic potential of targeting KRAS driver mutations

Tumor	Estimate new cases in 2017	Estimated death in 2017	Frequency of KRAS mutation	% of All KRAS mutations						
				G12A	G12D	G12R	G12C	G12S	G12V	G13D
Pancreas Cancer	53,670	43,090	**70%**	2	**51**	**12**	3	2	**30**	1
Colorectal	135,430	50,260	**36%**	7	**34**	1	9	5	**24**	19
Lung and Bronchus	222,500	115,870	**20%**	7	**17**	2	**42**	5	**20**	2
Endometrial	61,380	10,920	18%	11	**36**	0	9	2	**24**	15
Ovarian	22,440	14,080	14%	4	**41**	2	5	0	**37**	5
Prostate	161,360	26,730	7%	2	**22**	1	10	3	**35**	23

modified from Cosmic database and seer.cancer.gov

Supplementary Notes

Supplementary Note 1

Pt. 4238 IVS

We have employed our IVS approach on PBLs from 6 metastatic cancer patients harboring *KRAS* non-synonymous mutations in their tumors. We were able to detect and isolate reactive T cells targeting *KRAS* mutations from PBLs from three patients, Pt.4148, Pt.4171, and Pt.4238. The reactivities detected in the memory T subsets from the first two patients were presented in figures 3 and 4. Utilizing our IVS approach to apheresis samples from Pt. 4238, metastatic sigmoid colon adenocarcinoma patient harboring a G12D mutation in the excised tumor, enabled us to detect and isolate 6 TCRs targeting $KRAS^{G12D}$ (**Supplementary Figure 8**). Briefly, apheresis vials were thawed, rested overnight and sorted into CD4 and CD8 naïve, memory, and bulk subsets, six populations in total. Following, T cell subsets were stimulated for ten days with $KRAS^{G12D}$ 24mer, as described in the methods. At day ten the cells were co-cultured with autologous DCs pulsed with the peptide, at 3:1 T cells:DC ratio, and 4-1BB^{+} and/or OX40^{+} were sorted the next day. Sorted cells were expanded in REP protocol for 14 days and stimulated populations reactivities were tested in a co-culture assay in the presence of DCs that were pulsed with the 24mer mutated peptide. While no reactivity was observed in the bulk and naïve subsets a CD4 reactivity was detected in the memory population against the mutated but not the wild-type KRAS peptide (**Supplementary Figure 8A**). To isolate reactive TCRs we sorted memory CD4 cells upregulating T-cell activation markers, following co-culture with DCs pulsed with the mutated peptide, and performed scPCR and nested Sanger sequencing for the TCRs (**Supplementary Figure 8B**). Based on the sequencing results we synthesized, cloned and retrovirally transduced 13 TCRs into allogeneic PBLs from a healthy donor. Following, to

test the reactivity and the specificity of the TCRs we incubated the TCR-transduced cells with DCs pulsed with 10ug/ml of either the mutated KRAS and its counterpart WT peptide. Six TCRs, (TCR2, TCR3, TCR4, TCR6α2, TCR9, and TCR11α2) showed selective reactivity against the mutated peptide but not to the wild-type (**Supplementary Figure 8C**). To evaluate their avidity we co-incubated the allogeneic PBLs transduced with the reactive TCRs with DCs pulsed a serial dilution of the mutated and WT peptides. All six TCRs showed intermediate avidity with the comparable recognition at the same concentration range of mutated peptides as shown in **Supplementary Figure 8D.**

In summary, we were able to identify and isolate mutated *KRAS*-targeting T-cell receptors from memory T-cells subsets in three out of six patients using our IVS approach of memory cells. This technique will, potentially, allow us to construct a library of TCRs targeting shared oncogenes that can be used in cancer immunotherapy as “off-the-shelf” reagent. In an ongoing study, we are using our approach in an attempt to isolate TCRs targeting different shared oncogenes.

Supplementary Note 2

Pt. 4217 IVS

To further evaluate the IVS method we retrospectively tested our approach using PBLs isolated from a second metastatic colon cancer patient (patient 4217). This patient was also previously screened in our lab for the presence of neoantigen specific TIL and reactivities were found against 1 CD8 ($MUC4^{R4435S}$) and 2 CD4 ($RAD51B^{L202R}$ and $UEVLD^{F191V}$) epitopes (**Supplementary Figure 9**). Next, we sorted $CD8^{+}$ and $CD4^{+}$ memory (T_{CM}, T_{EM}, and T_{EMRA}) cells from Pt. 4217 PBMC and performed the IVS procedure with 3 TMG’s **(Supplementary data 1)** covering 48 out of the 170 neoepitopes identified by exome and RNA sequencing. The initial screen identify reactivity against TMG4 in the enriched memory compartement (**Supplementary Figures 3B, C**) while high background activity was observed in the bulk PBMC sample. No reactivity was detected in the $CD4^{+}$

cells (data not shown). To identify which mutated antigens were recognized in TMG-4 we co-cultured the enriched memory cells with autologous DCs that were individually pulsed with the mutated peptides encoded by TMG-4. As seen in **Supplementary Figure 3D**, the $MUC4^{R4435S}$ peptide was recognized by the enriched memory cells, and only the mutated peptide and not the WT was recognized (**Supplementary Figure 3E**).
