# Supplementary material for: Memory T cells targeting oncogenic mutations detected in peripheral blood of epithelial cancer patients
Source: Nat Commun. 2019 Jan 25;10:449. doi: 10.1038/s41467-019-08304-z (PMC6347629; doi:10.1038/s41467-019-08304-z)
Supplement: Supplementary file 9 — Reporting Summary [file 41467_2019_8304_MOESM9_ESM.pdf]

nature research

Corresponding author(s): Steven A Rosenberg

Reporting Summary

Nature Research wishes to improve the reproducibility of the work that we publish. This form provides structure for consistency and transparency in reporting. For further information on Nature Research policies, see Authors & Referees and the Editorial Policy Checklist.

Statistical parameters

When statistical analyses are reported, confirm that the following items are present in the relevant location (e.g. figure legend, table legend, main text, or Methods section).

n/a	Confirmed	
☐	☒	The exact sample size (*n*) for each experimental group/condition, given as a discrete number and unit of measurement
☐	☒	An indication of whether measurements were taken from distinct samples or whether the same sample was measured repeatedly
☐	☒	The statistical test(s) used AND whether they are one- or two-sided<br>*Only common tests should be described solely by name; describe more complex techniques in the Methods section.*
☐	☒	A description of all covariates tested
☒	☐	A description of any assumptions or corrections, such as tests of normality and adjustment for multiple comparisons
☐	☒	A full description of the statistics including central tendency (e.g. means) or other basic estimates (e.g. regression coefficient) AND variation (e.g. standard deviation) or associated estimates of uncertainty (e.g. confidence intervals)
☒	☐	For null hypothesis testing, the test statistic (e.g. *F*, *t*, *r*) with confidence intervals, effect sizes, degrees of freedom and *P* value noted<br>*Give P values as exact values whenever suitable.*
☒	☐	For Bayesian analysis, information on the choice of priors and Markov chain Monte Carlo settings
☒	☐	For hierarchical and complex designs, identification of the appropriate level for tests and full reporting of outcomes
☒	☐	Estimates of effect sizes (e.g. Cohen's *d*, Pearson's *r*), indicating how they were calculated
☐	☒	Clearly defined error bars<br>*State explicitly what error bars represent (e.g. SD, SE, CI)*

Our web collection on statistics for biologists may be useful.

Software and code

Policy information about availability of computer code

Data collection	BD Diva, CTL immunospot
Data analysis	GraphPad Prism version 6, FlowJo version 10, Microsoft Excel 365 ProPlus

For manuscripts utilizing custom algorithms or software that are central to the research but not yet described in published literature, software must be made available to editors/reviewers upon request. We strongly encourage code deposition in a community repository (e.g. GitHub). See the Nature Research guidelines for submitting code & software for further information.

Data

Policy information about availability of data

All manuscripts must include a data availability statement. This statement should provide the following information, where applicable:

- Accession codes, unique identifiers, or web links for publicly available datasets
- A list of figures that have associated raw data
- A description of any restrictions on data availability

Data availability statement is included in the manuscript

Field-specific reporting

Please select the best fit for your research. If you are not sure, read the appropriate sections before making your selection.

☒ Life sciences ☐ Behavioural & social sciences ☐ Ecological, evolutionary & environmental sciences

For a reference copy of the document with all sections, see nature.com/authors/policies/ReportingSummary-flat.pdf

Life sciences study design

All studies must disclose on these points even when the disclosure is negative.

Sample size	Sample size was selected based on the number of patients for whom we had gathered complete data sets.
Data exclusions	Not applicable
Replication	All attempts for replication of the results, where applicable, were successful.
Randomization	not applicable
Blinding	not applicable

Reporting for specific materials, systems and methods

Materials & experimental systems

n/a	Involved in the study
☐	☒ Unique biological materials
☐	☒ Antibodies
☐	☒ Eukaryotic cell lines
☒	☐ Palaeontology
☒	☐ Animals and other organisms
☐	☒ Human research participants

Methods

n/a	Involved in the study
☒	☐ ChIP-seq
☐	☒ Flow cytometry
☒	☐ MRI-based neuroimaging

Unique biological materials

Policy information about availability of materials

Obtaining unique materials	Individualized cellular immunotherapy, limited research material. Materials available for research institutions with MTA or for-profit companies with license.

Antibodies

Antibodies used	The following titrated anti-human antibodies were used for cell surface staining: CCR7- BB515 (cat. 565869, 1:12.5 dilution) or FITC (cat. 561271, 1:12.5 dilution), CD45RO-PE-Cy7 (cat. 560608, 1:7 dilution) or APC (cat.559865, 1:7 dilution), CD45RA-APC (cat. 561210, 1:7 dilution) or BD Horizon™ V450 (cat. 560362, 1:7 dilution), CD62L-PE (cat. 555544, 1:7 dilution), CD4-PE (cat. 566679, 1:100 dilution), FITC (cat. 56561005, 1:100 dilution), BV605 (cat. 562659, 1:100 dilution) , CD3- AF700 (cat. 561027, 1:100 dilution) or APC-H7 (cat. 560176, 1:100 dilution), CD4-FITC, PE, PE-Cy7, APC-H7 (clone: SK3), CD8-PE-Cy7 (cat. 335787, 1:100 dilution), OX40-PE-Cy7 (cat. 563663, 1:7 dilution) or FITC (cat. 555837, 1:7 dilution), 4-1BB-APC (cat. 550890, 1:7 dilution). All antibodies were from BD Biosciences. For MHC blocking assays, the following antibodies were used: pan-class-I (clone: W6/32, produced from hybridoma and used at 1:20 dilution), pan-class-II (BD pharmingen, cat. 555556, 1:20 dilution), HLA-DR (clone: HB55, produced from hybridoma and used at 1:20 dilution), HLA-DP (Leinco, cat.H127, used at 15 mg/ml), and HLA-DQ (BD pharmingen, cat. 555562, 1:20 dilution). For cells stimulation purified anti CD3 was used (Miltenyi Biotech, cat.130-093-387, used at 30 ng/ml).
Validation	Validation was done by the vendor

Eukaryotic cell lines

Policy information about cell lines

Cell line source(s)	HEK293GP: Surgery Branch, NCI<br>COS7: ATCC<br>FA6-2: Surgery Branch, NCI<br>Mia-PaCa: ATCC<br>NCI-H441: ATCC<br>SW480: ATCC<br>SW900: ATCC
Authentication	HEK293GP: by DNA fingerprinting for human gal/pol expression was authenticated by production of transient vector production.<br>FA6-2, Mia-PaCa, NCI-H441, SW480, SW900: The cell lines were authenticated by HLA genotyping and KRAS mutation status, and transduced with retrovirus encoding HLA-A*11:01.
Mycoplasma contamination	All tested negative for mycoplasma contamination
Commonly misidentified lines<br>(See ICLAC register)	N/A

Human research participants

Policy information about studies involving human research participants

Population characteristics	Patient samples were derived from patients enrolled on clinical protocols (Clinicaltrials.gov: NCT01174121, NCT02621021, and NCT01993719) approved by the institutional-review board (IRB) of the National Cancer Institute (NCI)
Recruitment	Patient samples were derived from patients enrolled on clinical protocols (Clinicaltrials.gov: NCT01174121, NCT02621021, and NCT01993719) approved by the institutional-review board (IRB) of the National Cancer Institute (NCI)

Flow Cytometry

Plots

Confirm that:

☒ The axis labels state the marker and fluorochrome used (e.g. CD4-FITC).

☒ The axis scales are clearly visible. Include numbers along axes only for bottom left plot of group (a 'group' is an analysis of identical markers).

☒ All plots are contour plots with outliers or pseudocolor plots.

☒ A numerical value for number of cells or percentage (with statistics) is provided.

Methodology

Sample preparation	Described in materials and methods
Instrument	BD Aria, LSR-II
Software	BD Diva
Cell population abundance	Described in manuscript
Gating strategy	Described in materials and methods

☒ Tick this box to confirm that a figure exemplifying the gating strategy is provided in the Supplementary Information.
